# The data not collected on community forestry

**DOI:** 10.1111/cobi.12732

**Published:** 2016-06-15

**Authors:** Reem Hajjar, Johan A. Oldekop, Peter Cronkleton, Emily Etue, Peter Newton, Aaron J.M. Russel, Januarti Sinarra Tjajadi, Wen Zhou, Arun Agrawal

**Affiliations:** ^1^ School of Natural Resources and EnvironmentThe University of Michigan440 Church StreetAnn ArborMI48109U.S.A.; ^2^ School of BiologyNewcastle UniversityNewcastle upon TyneNE1 7RUU.K.; ^3^ Center for International Forestry Research (CIFOR)Av. La Molina 1895La MolinaLimaPeru; ^4^ The Center for People and Forest (RECOFTC)BangkokThailand; ^5^ Environmental Studies Program, Sustainability, Energy and Environment ComplexUniversity of ColoradoBoulderCO 80303U.S.A.; ^6^ CIFORSitu Gede, Sindang Barang, Bogor (Barat) 16115Indonesia

**Keywords:** biophysical factors, community‐managed forests, institutional arrangements, markets, socioeconomic characteristics, systematic map, arreglos institucionales, bosques administrados por comunidades, características socioeconómicas, factores biofísicos, mapa sistemático, mercados

## Abstract

Conservation and development practitioners increasingly promote community forestry as a way to conserve ecosystem services, consolidate resource rights, and reduce poverty. However, outcomes of community forestry have been mixed; many initiatives failed to achieve intended objectives. There is a rich literature on institutional arrangements of community forestry, but there has been little effort to examine the role of socioeconomic, market, and biophysical factors in shaping both land‐cover change dynamics and individual and collective livelihood outcomes. We systematically reviewed the peer‐reviewed literature on community forestry to examine and quantify existing knowledge gaps in the community‐forestry literature relative to these factors. In examining 697 cases of community forest management (CFM), extracted from 267 peer‐reviewed publications, we found 3 key trends that limit understanding of community forestry. First, we found substantial data gaps linking population dynamics, market forces, and biophysical characteristics to both environmental and livelihood outcomes. Second, most studies focused on environmental outcomes, and the majority of studies that assessed socioeconomic outcomes relied on qualitative data, making comparisons across cases difficult. Finally, there was a heavy bias toward studies on South Asian forests, indicating that the literature on community forestry may not be representative of decentralization policies and CFM globally.

## Abstract (es)

Los Datos No‐Colectados de la Silvicultura Comunitaria

**Resumen**

Quienes practican la conservación y el desarrollo promueven la silvicultura comunitaria como una manera de conservar los servicios ambientales, consolidar los derechos a los recursos y reducir la pobreza. Sin embargo, los resultados de la silvicultura comunitaria han sido mixtos; muchas iniciativas fallaron en la adquisición de los objetivos planeados. Existe una literatura amplia sobre los arreglos institucionales de la silvicultura comunitaria pero ha habido muy pocos esfuerzos por examinar el papel que los factores socioeconómicos, de mercado y biofísicos tienen en la formación de las dinámicas de cambio de cobertura de suelo y en los resultados del sustento individual y colectivo. Revisamos sistemáticamente la literatura revisada por pares sobre la silvicultura comunitaria para examinar y cuantificar los vacíos de conocimiento existentes en la literatura de silvicultura comunitaria en relación con estos factores. En la revisión de 697 casos de manejo comunitario de bosques, extraídos de 267 publicaciones revisadas por pares, encontramos tres tendencias clave que limitan el entendimiento de la silvicultura comunitaria. Primero, encontramos vacíos sustanciales de datos que conectaban las dinámicas poblacionales, las fuerzas de mercado y las características biofísicas con los resultados ambientales y de sustento. Segundo, la mayoría de los estudios se enfocaron en resultados ambientales y la mayoría de los estudios que evaluaron los resultados socioeconómicos dependieron de datos cualitativos, lo que hizo que las comparaciones entre casos se complicaran. Finalmente, hubo un sesgo notable hacia los estudios en los bosques del sur de Asia, lo que indica que la literatura sobre la silvicultura comunitaria puede no ser representativa ni de las políticas de descentralización ni del manejo comunitario de bosques a nivel global.

## Introduction

Decentralization of natural resource management is central to a rights‐based approach to conservation and sustainable development (UN 2015). Decentralization of forest management has been a major trend in global forest governance since the 1980s (Agrawal et al. 2008), and international conservation and development practitioners have increasingly promoted community‐managed forests as a way to enhance sustainable forest use, consolidate rights over traditional lands and resources, and reduce rural poverty (Bray et al. 2003; Molnar et al. 2008). Case studies from around the world show that community forestry has the potential to deliver economic, sociocultural, and ecological benefits to local communities and to improve sustainable forest use and livelihood outcomes (Pagdee et al. 2006; Bowler et al. 2012). Despite successes, outcomes generally have been mixed; many initiatives have failed to achieve intended objectives (Edmunds & Wollenberg 2003; Oyono 2005; Pokorny 2009).

To gain a better understanding of livelihood and forest outcomes, several studies have focused on the effects of institutional arrangements associated with community forests, examining, inter alia, design principles of community‐based resource management (Ostrom 1999; Gibson et al. 2000) and tenure and institutional settings and their influences on forest management decision making, local livelihoods, forest biodiversity, and carbon storage (Agrawal & Gibson 1999; Bray et al. 2003; Chhatre & Agrawal 2009; Persha et al. 2011). A number of meta‐analyses have aimed to determine factors that lead to success of community forestry (Pagdee et al. 2006; Oldekop et al. 2010; Baynes et al. 2015), including a review of the links between community tenure and forest condition (Seymour et al. 2014) and an examination of whether formal community forest management (CFM) has been more effective than either no CFM or than alternative tenure arrangements (Bowler et al. 2012).

Previous analyses of community‐forest outcomes have focused predominantly on limited subsets of key institutional and socioeconomic variables. However, the relative effects of different community‐forestry arrangements and the role of social, political, economic, and biophysical factors in shaping outcomes of community forestry remain poorly understood. Elucidating these relationships, which affect livelihood decisions and forest dynamics at various scales, is key for providing a strong evidence base for the design and implementation of improved decentralized policies on natural resource management.

To identify further areas of research and assess whether the literature allows for meaningful conclusions to be made about the broader set of community‐forestry arrangements and the social, political, economic, and biophysical drivers of outcomes of community forestry, it is important to first examine and quantify existing knowledge gaps related to these drivers and outcomes. Thus, we compiled over 2 decades of peer‐reviewed research on community forestry and examined the frequency and types of information collected and published regarding community forestry around the world.

## Methods

Our method is detailed in Newton et al. (2015). Here, we provide a brief overview of our methods, elaborating only when they evolved from those published previously.

### Framing the Study

We expanded the PICO (population, intervention, comparator, outcomes) framework, traditionally used to frame systematic review questions, search terms, and study inclusion criteria (Counsell 1997; CEE 2013), to include a broader set of contextual factors (PICOC) that might act as mediators of arrangements of community forestry (Petticrew & Roberts 2006). Our population of interest was individual forest units and the communities of people managing them. We defined *community forest* as a forest being shared among at least 3 households (as defined by the International Forestry Resources and Institutions [IFRI; 2012] research network). We focused on community forests in less industrialized nations in Latin American, African, and Asia‐Pacific regions, which is where the majority of community forests are located (RRI 2013). Cases of afforestation (except enrichment planting) or exotic species plantations were not included to ensure comparability among environmental outcomes across natural forests.

Our intervention of interest was community forestry, broadly defined as forest use and governance arrangements under which the rights, responsibilities, and authority for forest management rest, at least in part, with local communities. We included both traditional and endogenous community‐forestry initiatives undertaken by forest user groups as well as initiatives introduced by external actors (e.g., nongovernmental organizations or governments). The latter includes project‐based initiatives and policies aimed at decentralizing forest management or reforming land or resource tenure. We examined variations across temporal and spatial dimensions (differences over time and across locations).

**Figure 1:**
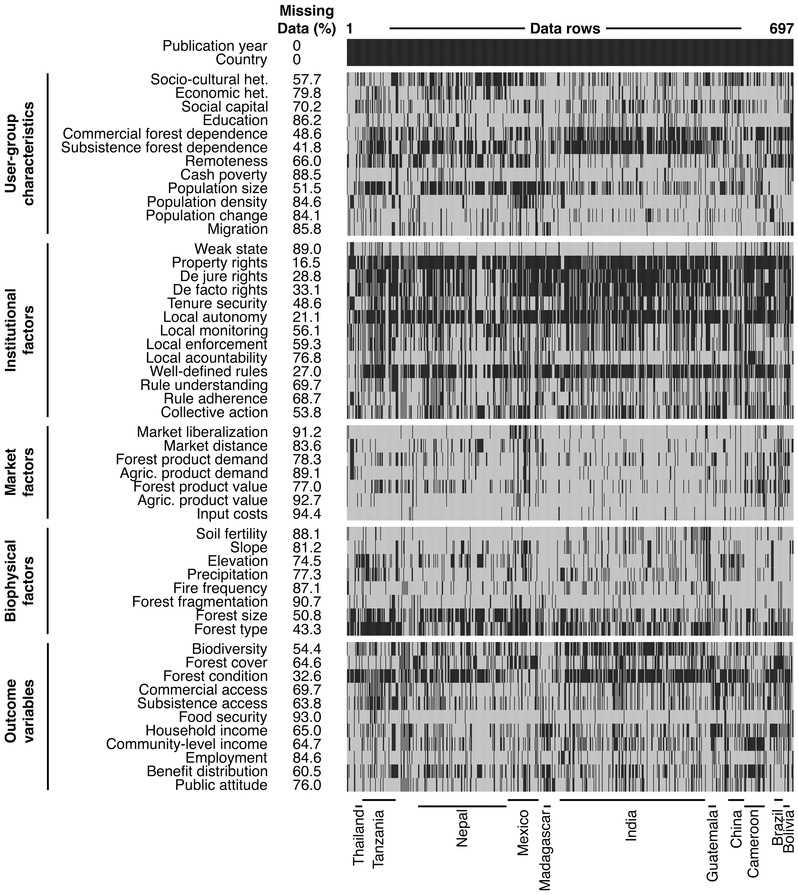
Data map indicating variables extracted from 697 cases of community forestry (black, recorded data; gray, missing data). Variables are thematically grouped (user‐group characteristics, institutional factors, market factors, biophysical factors, and outcome variables), and data rows are grouped by countries with 10 cases or more.

Our outcomes of interest were environmental and livelihood indicators that represent key aims of community‐managed forest interventions (Charnley & Poe 2007; Persha et al. 2010). These included measures of environmental change related to forest cover, forest condition, and biodiversity and livelihood change related to access to forest resources for commercial or subsistence use, food security, household and community income, employment, and benefit distribution. We also examined 40 variables (contextual factors) representing sources of variation associated with forest outcomes, including user‐group socioeconomic and demographic characteristics, forest‐ and agriculture‐related market factors, institutional factors related to forest management, and biophysical factors (Fig. 1). We created this list through a preliminary review of 35 frequently cited articles on community forestry and forest‐cover change (Supporting Information) (identified through a search on Google Scholar and Web of Science). We modified or added variables during the testing phase of the data‐extraction protocol.

### Search Strategy and Inclusion Criteria

We performed a series of Boolean searches between May and October 2014 in 2 publication databases (Web of Knowledge and CAB Abstracts). The 76 search terms and search strings are listed in Newton et al. (2015).

We included only publications in English, met criteria for population and intervention definitions as outlined in the PICOC, contained data on any of the environmental or livelihood outcome metrics, and contained at least one of the contextual variables. Articles also had to be published in a peer‐reviewed journal. This excluded an extensive gray literature on community forests, but it ensured that data were less likely to be double‐counted if published in different formats and studies had undergone an independent, peer‐review process prior to publication. An article needed to contain new primary data to be included; review papers and meta‐analyses were excluded.

We screened papers for inclusion criteria in 3 stages: first, titles and abstracts; second, full texts; and third, availability of data for extraction (see “Data Extraction”). To ensure interrater consistency, we performed free‐marginal kappa analyses (Randolph 2005) at the beginning of each screening stage on a subset of randomly selected studies until the screening and extraction teams reached acceptable levels of agreement (κ > 0.60).

### Data Extraction

We extracted quantitative and qualitative data on contextual and outcome variables for each case of community forestry presented in each paper that passed through the entire screening processes. For publications presenting multiple cases of community‐managed forests and to the extent possible, we extracted data separately for each case that represented a unique community forest.

## Results

From our initial pool of 15,879 articles, we extracted data from 267 papers. From these papers, we identified 735 cases of community‐managed forests, yielding data on a total of 697 cases for subsequent analysis once duplicate cases focusing on the same sites were consolidated.

### Variables

The extent to which the reviewed papers analyzed or reported on particular variables varied enormously (Fig. 1). Institutional factors were the most frequently included and market factors the least. Less than 30% of the cases reported on market characteristics, user‐group characteristics, and biophysical attributes other than forest type and size. Rights, existence of well‐defined local rules, and levels of autonomy were most frequently included (≥70% of cases), whereas other institutional factors, such as strength of nonlocal government institutions, stakeholder understanding of and adherence to local rules, and accountability of local leaders to their community were included far less frequently (<30% of cases). In terms of user‐group characteristics, studies most frequently focused on levels of forest subsistence (51–58%) and sociocultural heterogeneity within groups (42%) and focused less on basic demographics such as population density and change, migration, education, and cash poverty (all approximately 15%). Few studies attended to biophysical factors other than forest type and size (56% and 49%, respectively).

### CFM Outcomes

We considered various environmental and livelihood outcomes reported in the CFM literature. Forest condition was the most frequently reported outcome variable (68%). All other outcomes were reported in <50% of the cases. Most of the livelihood outcomes were reported in 30–40% of the cases. Despite common perceptions that decentralizing forest management to communities increases local employment (e.g., Bray et al. 2003; Charnley & Poe 2007), relatively few studies (15% of cases) measured changes in employment levels. Very few studies considered the implications of CFM on food security (7%). Public attitudes toward CFM were reported in 25% of the cases.

Environmental outcomes were measured using quantitative approaches in 56% of cases. Livelihood outcomes, in contrast, were rarely (24%) measured using quantitative surveying techniques, except for income and benefit distribution. Rather, respondent perceptions were the typical means through which researchers assessed outcomes. For example, although access to subsistence forest was one of the most often recorded livelihood outcomes, few researchers used surveys to record changes in forest access and usage by households.

### Regional Distribution

**Figure 2:**
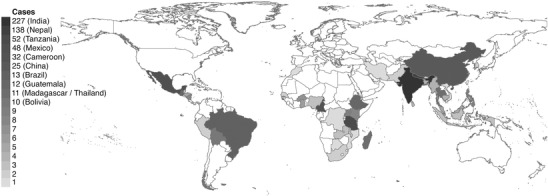
Number of cases of community forestry in individual countries within the final sample of 267 peer‐reviewed papers. The 11 countries with ≥10 cases of community forestry are shown (India and Nepal, 52% of cases).

Most cases were from South Asia (Fig. 2); community‐managed forests in India and Nepal accounted for 33% and 20% of all published cases, respectively. In Latin America, Mexico dominated with 7% of all cases. In Africa, Tanzania (8%) and Cameroon (5%) had the highest number of cases. In East and Southeast Asia, the region with the fewest studies, 4% of cases were located in China.

## Discussion

Our review provides a unique overview of the evidence available in the peer‐reviewed literature on community forestry globally and contributes to the wider natural resources management decentralization literature in 3 significant ways.

First, we found significant gaps in understanding of the role of population dynamics, market forces, and biophysical factors as drivers of environmental and livelihood outcomes of community forestry. Given the large number and variety of variables on which we collected information, it was not surprising that most studies reported on <50% of all variables. Of particular importance, however, is the frequency with which some groups of factors were absent. Population dynamics and market and biophysical factors (besides forest type and size) were rarely considered. Much of the literature on CFM continues to focus on institutional factors (Fig. 1), despite the fact that population, market, and biophysical factors also affect the dynamics of forest and land‐cover change (Geist & Lambin 2002; Agrawal & Chhatre 2006; Meyfroidt & Lambin 2011; Rudel et al. 2012), as well as local livelihood decisions and the dynamics and outcomes of collective resource management (Agrawal & Yadama 1997; Agrawal 2001; Agrawal & Chhatre 2006; Oldekop et al. 2013). It is possible that some of the gaps we identified may not be as large as they appear. Some researchers may have chosen to study certain factors with an awareness that other factors would be closely correlated (e.g. altitude, slope, and precipitation). Nonetheless, the absences highlighted in our data set point to significant and continuing gaps in understanding of the factors driving community‐forestry outcomes.

Second, livelihood outcomes have been assessed primarily with qualitative methods. Although existing qualitative studies provide useful insights into the kinds of socioeconomic impacts community forestry can have, there is an urgent need to complement these studies with nonperception‐based measures to make comparative assessments of intervention outcomes across sites and help establish baselines for longitudinal studies.

Finally, the CFM literature was heavily biased toward cases in South Asia (predominantly in India and Nepal) and thus was likely not representative of decentralization and community forestry globally. No global data sets on community forests exist, and even national inventories are rare. However, it is possible that South Asia and Africa are overrepresented in the literature, particularly given trends in increasing incidence of community tenures in several Latin American and Southeast Asian countries. Although this may be partly an artifact of our focus on the English‐language literature, more forests are under community control or ownership in Latin America than in Africa (36% and 6%, respectively) (RRI 2013). In absolute terms, the area of forests in Latin America under community control is an order of magnitude larger than in Africa or South Asia (225.75, 22.89, and 28.27 million ha, respectively [RRI 2013]), yet cases from Africa represent 25% of the reported analyses in the literature, and India and Nepal represent >50%. For a more representative global understanding of outcomes of CFM, future research needs to address these 3 critical gaps.

## Supporting information

Information on the variables chosen for inclusion in the analysis (Appendix S1) is available online. The authors are solely responsible for the content and functionality of these materials. Queries (other than absence of the material) should be directed to the corresponding author.
