# Supplementary material for: The data not collected on community forestry
Source: Conserv Biol. 2016 Jun 15;30(6):1357–62. doi: 10.1111/cobi.12732 (PMC5111782; doi:10.1111/cobi.12732)
Supplement: Supplementary file 1 — Information on the variables chosen for inclusion in the analysis (Appendix S1) is available online. The authors are solely responsible for the content and functionality of these materials. Queries (other than absence of the material) should be directed to the corresponding author. [file COBI-30-1357-s001.docx]

**Appendix S1**

| *Category* | *Variables measured* | *Justification for inclusion* | |
| --- | --- | --- | --- |
| User-group characteristics | socio-cultural heterogeneity, economic heterogeneity, social capital, education, forest dependence commercial,  forest dependence subsistence, remoteness, cash poverty | Forest outcomes are associated with user groups’ historical experience with resource management, size of user group, level of social capital among members, strength of leaders, and heterogeneity of the group with respect to wealth, ethnicity, or religion (Agrawal 2001). |  |
| Institutional characteristics | regulation enforcement at higher level, property rights, de jure rights, de facto rights, tenure security, autonomy, local rules, rule understanding, rule adherence, monitoring, enforcement, accountability, collective action | Forest outcomes are associated with institutional arrangements, especially those concerned with rights to create and enforce rules and to hold officials accountable (Ostrom 1990, Baland & Platteau 1996). | |
| Market characteristics | liberalization, market distance, market demand, input cost, product value, other policies | Forest outcomes are associated with demand and accessibility to markets and differing land use wage rates which influences the incentive and capacity to log forested areas (Chomitz & Gray 1996, Cropper et. al 2001, Deininger & Minten 2002). | |
| Biophysical characteristics | soil fertility, elevation, slope, forest type, fragmentation, precipitation, fire, forest size, other contextual factors | Forest outcomes are associated with how much a forested area is biophysically suitable for other uses (Andam et. al 2008, Cropper et. al 2001, Deininger & Minten 2002). | |
| Demographic characteristics | population density, population change, migration | Forest outcomes are associated with change in human populations due to demand for food, timber, and land for agriculture or pasture (Allen & Barnes 1989, Cropper et. al 2001). | |

**TABLE 1: Variables affecting environmental and socio-economic outcomes of community-managed forests**

References:

Agrawal, A. (2001) Common property institutions and sustainable governance of resources. World Development 29, 1649-1672.

Allen, J.C., Barnes, D.F. (1985) The causes of deforestation in developing countries. Annals of the association of American Geographers 75, 163-184.

Andam, K.S., Ferraro, P.J., Pfaff, A., Sanchez-Azofeifa, G.A., Robalino, J.A. (2008) Measuring the effectiveness of protected area networks in reducing deforestation. Proceedings of the National Academy of Sciences 105, 16089-16094.

Baland, J.-M., Platteau, J.-P. (1996) Halting degradation of natural resources: is there a role for rural communities? Food & Agriculture Org.

Chomitz, K.M., Gray, D.A. (1996) Roads, land use, and deforestation: a spatial model applied to Belize. The World Bank Economic Review 10, 487-512.

Cropper, M., Puri, J., Griffiths, C. (2001) Predicting the location of deforestation: The role of roads and protected areas in North Thailand. Land Economics 77, 172-186.

Deininger, K., Minten, B. (2002) Determinants of deforestation and the economics of protection: An application to Mexico. American Journal of Agricultural Economics 84, 943-960.

Ostrom, E. (1990) Governing the commons: The evolution of institutions for collective action. Cambridge University Press.
